# Endothelial cell plasticity at the single-cell level

**DOI:** 10.1007/s10456-021-09797-3

**Published:** 2021-06-01

**Authors:** Alessandra Pasut, Lisa M. Becker, Anne Cuypers, Peter Carmeliet

**Affiliations:** 1 Laboratory of Angiogenesis and Vascular Metabolism, Vesalius Research Center, VIB, K.U.Leuven, Campus Gasthuisberg, Herestraat 49, B-3000 Leuven, Belgium; 2 Laboratory of Angiogenesis and Vascular Metabolism, Department of Oncology, KU Leuven, Leuven, Belgium; 3 Laboratory of Angiogenesis and Vascular Heterogeneity, Department of Biomedicine, Aarhus University, 8000 Aarhus C, Denmark; 4 State Key Laboratory of Ophthalmology, Zhongshan Ophthalmic Center, Sun Yat-Sen University, Guangzhou, Guangdong P.R. China

**Keywords:** Endothelial cells, Single-cell analyses, Angiogenesis, Tissue repair, Tumorigenesis, Metabolism

## Abstract

The vascular endothelium is characterized by a remarkable level of plasticity, which is the driving force not only of physiological repair/remodeling of adult tissues but also of pathological angiogenesis. The resulting heterogeneity of endothelial cells (ECs) makes targeting the endothelium challenging, no less because many EC phenotypes are yet to be identified and functionally inventorized. Efforts to map the vasculature at the single-cell level have been instrumental to capture the diversity of EC types and states at a remarkable depth in both normal and pathological states. Here, we discuss new EC subtypes and functions emerging from recent single-cell studies in health and disease. Interestingly, such studies revealed distinct metabolic gene signatures in different EC phenotypes, which deserve further consideration for therapy. We highlight how this metabolic targeting strategy could potentially be used to promote (for tissue repair) or block (in tumor) angiogenesis in a tissue or even vascular bed-specific manner.

## Introduction

The vascular endothelium is heterogeneous as it dynamically engages in different functions, influenced by the physiological needs, energetic demands, and distinct conditions of different tissues. These functions include the delivery and exchange of oxygen/nutrients, the regulation of blood flow dynamics (vasoregulation) and hemostasis, the maintenance of tissue–blood barrier functions (trafficking of immune cells, transport of proteins), and tissue-specific angiocrine functions, among others [1–3]. The ability to interrogate the transcriptome at the single-cell level has improved our understanding of EC heterogeneity. Indeed, recent EC-centered single-cell studies revealed a remarkable transcriptional heterogeneity of ECs across vascular beds and tissues, leading to the generation of new concepts and identification of previously unrecognized EC subtypes [4–13].

While we recognize that mural and smooth muscle cells are also important players of the vasculature, we mainly focus here on EC heterogeneity and highlight key examples of EC plasticity, including (i) the diversification/specification of the vascular endothelium in distinct subtypes and (ii) EC plasticity (the ability of quiescent ECs to acquire an angiogenic phenotype). Endothelial-to-mesenchymal transition (EndMT) [14] and the specification of the lymphatic endothelium are other examples of EC plasticity, previously highlighted in recent reviews [14–18].

Focusing primarily on data emerging from single-cell studies, we highlight key mechanisms driving EC plasticity in health and disease, including transcription factor dynamics, epigenetic or tissue-specific cues, and metabolic transcriptome plasticity [19]. We discuss how the vascular endothelium is endowed with different functions that are carried out by specialized EC subtypes, capable of switching phenotypes partially through reprogramming their metabolism. Identification of functionally relevant ECs and the instructive signals that regulate their plasticity will be instrumental to develop novel approaches for anti- or pro-angiogenic therapeutic strategies [20–22]. Here, without an intention to provide an all-encompassing historic overview, we revisit classic and emerging concepts of EC heterogeneity in development and disease, focusing also on recent single-cell data.

## Endothelial cell heterogeneity and plasticity

### Diversification of endothelial cells

The specification of an arterial, capillary, venous, and lymphatic EC phenotype is a critical event for the development of the vasculature [2, 13, 23–25]. Lineage tracing experiments combined with gene reporter activity were among the first to proof the ability of ECs to interconvert between arterial and venous phenotypes. These experiments showed a requirement for VEGF, Notch, and COUP-TF2 in the specification of vessel identity, with higher expression of Notch and VEGF specifying the arterial fate *versus* COUP-TF2 inhibiting Notch signaling in vein ECs [23, 24, 26–28].

**Fig. 1:**
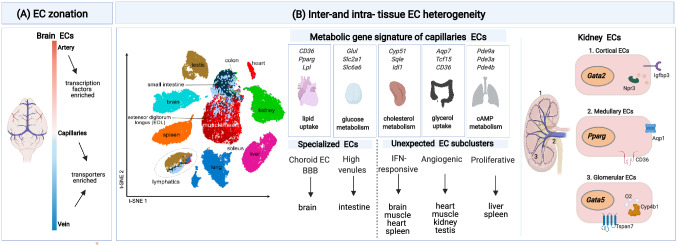
EC heterogeneity: **A** Illustration of EC heterogeneity in the brain along the arteriovenous axis (cellular zonation), as unveiled by scRNA-seq adapted from 10. Color-coded bar indicates the gradual changes in gene expression along the arteriovenous axis. Transcription factor genes are enriched in arteries, whereas genes encoding transporters are increased in capillaries and veins. **B** Single-cell transcriptomic studies of ECs in adult tissues revealed both intra- and inter-tissue heterogeneity. Left: t-SNE plot showing that ECs from some tissues cluster together based on transcriptomic similarities adapted from 4. Middle: Depiction of tissue-specific metabolic gene signatures of capillary ECs, specialized tissue-restricted EC phenotypes (choroid ECs in the brain; high endothelial venule (HEV)-like ECs in the intestine 4), and unexpected EC phenotypes, as identified by scRNA-seq studies. Unexpected EC phenotypes include IFN-activated ECs (found in brain, muscle, heart, and spleen), angiogenic ECs (found in heart, muscle, kidney, and testis), and proliferating ECs (found in liver and spleen). Right: EC heterogeneity in the kidney, determined by topological position, illustrated by some key enriched genes expressed by cortical, medullary, and glomerular ECs. O2: oxygen

Technological advancements in single-cell -omics studies expanded the opportunities to study EC heterogeneity. To date, single-cell atlases have been constructed from nearly every organ, but only a few of those studies, in particular only when combined with EC enrichment strategies via genetic labeling or use of an EC surface marker, exhibit sufficient power to discover novel EC phenotypes [4–8, 10, 12, 29–31]. A pioneering single-cell transcriptomics study identified the gene expression signature of putative arterial, venous, and capillary ECs in the murine brain, revealing “EC zonation” of these signatures characterized by gradual changes in gene expression [10]. When examining the molecular functions of the most highly expressed genes in each subtype, the authors observed that transcription factors were over-represented in arterial ECs, while transporter expression dominated in capillary and venous ECs [10] (Fig. 1A).

Earlier bulk transcriptomics studies revealed that ECs from different tissues have distinct transcriptomic signatures [32]. These findings have now been expanded by single-cell RNA sequencing studies. In one of the (to date) largest follow-up single-cell RNA sequencing studies using ECs, isolated from 11 adult healthy murine tissues, up to 78 EC populations were identified [4]. Several key insights were deduced from this study [4]. First, ECs from some tissues clustered together and their transcriptomes were more similar to each other, likely because their vasculature shared biological processes (Fig. 1B). For instance, brain and testis ECs resembled each other transcriptomically because they share a tight blood–tissue barrier, while small and large intestinal ECs share a vascular–gut barrier, etc. Second, compared to arterial, venous, and lymphatic ECs, which share multiple common markers across tissues and express few tissue-specific markers (suggesting relative transcriptomic stability), capillary ECs express fewer common markers, shared across tissues, and instead are characterized by tissue-specific markers, likely reflecting organ-specific metabolic and physiological needs [4]. In addition to the traditional (artery, capillary, vein, lymphatic) EC phenotypes, tissue-restricted and novel EC phenotypes were identified (proliferating capillaries in liver and spleen; angiogenic ECs in heart, muscle, kidney, and testis; IFN-activated ECs in brain, muscle, heart, and spleen) (Fig. 1B) [4, 6]. Third, ECs in vascular beds (arteries, capillaries, veins) transcriptomically resemble each other across tissues [4, 20]. Finally, not the position in the vascular tree, but the tissue type primarily contributes to EC heterogeneity [4, 9–11] (Fig. 1B).

Other analyses, such as re-analysis of single-cell RNA sequencing of ECs from the Tabula Muris consortium [8] and microarray analyses, reported similar findings of tissue-specific gene expression in ECs [8]. Pathway analysis of the most differentially expressed genes (DEG) in ECs across tissues showed that while some pathways are common to all ECs irrespective of the tissue of origin, tissue-specific preferences dictate which particular set of genes is expressed in ECs in any given tissue [4, 20], further highlighting tissue-specific EC specialization. An example is represented by brain ECs, which are heterogeneous (with EC subtypes unique to the brain, such as the choroid plexus ECs) compared to ECs from other tissues (heart and muscle) [4, 10, 20, 32]. In addition, even within the same organ, ECs are transcriptionally heterogenous depending on their topological (compartment-specific) position. For example, in the kidney, cortical, medullary, and glomerular ECs are transcriptionally distinct. Cortical ECs express high levels of *Ifgbp3* and *Npr3* and the transcription factor *Gata2*, whereas medullary ECs are enriched for *Pparg* and the fatty acid transporter *CD36* and *Aqp1* (the latter can only be found in a subset of medullary ECs). Lastly, glomerular ECs upregulate the expression of *Gata5*, *Tspan7*, and *Cyp4b1* (among other genes) (Fig. 1B). Within the same compartment, distinct subtypes of ECs were identified including capillary renal ECs with an angiogenic signature and renal vein and capillary ECs subtypes with an IFN signature [9]. The microvasculature of the liver also represents an additional example of tissue zonation, recently discussed elsewhere [11].

**Fig. 2:**
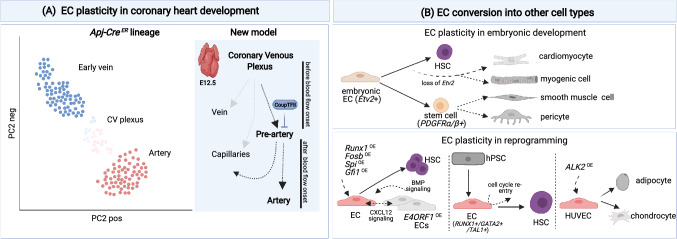
EC plasticity: **A** Randomized principle component analysis (rPCA) plot of single ECs in the sinus venous from *Apj-Cre* mice intercrossed with *Rosa*^*mTmG*^ reporter mice (to trace cells from the sinus venous), revealed clusters of early vein, coronary venous (CV), and artery ECs adapted from 12. The model proposes that during embryonic development (E12.5), ECs in the coronary venous plexus can adopt a pre-arterial fate before the onset of blood flow and eventually differentiate into coronary arterial ECs. CoupTFII inhibits pre-arterial to arterial EC differentiation. A subset of pre-arterial ECs differentiates into capillaries, while a fraction of ECs from the coronary venous plexus is also capable of differentiating into capillaries. **B** Top panel: Examples of EC plasticity during development. Embryonic ECs (expressing Etv2; key regulator of the endothelial/hematopoietic axis) are capable of differentiating into cardiac and skeletal muscle (myogenic) cells when Etv2 expression is silenced. In the presence of Etv2, embryonic ECs can differentiate into hematopoietic stem cells (HSCs). Embryonic ECs can also transition into stem-like cells (Pdgfrα/β + progenitors) that can further differentiate into mural cells (pericytes and smooth muscle cells) in the heart. Bottom panel: Examples of EC plasticity in different settings of reprogramming. Left, Adult ECs can be reprogrammed by transcription factor overexpression (*Fosb*, *Gfi1*, *Runx1*, *Spi1*) to differentiate into competent hematopoietic stem cells. ECs overexpressing E4ORF1 (presumed surrogates of niche cells) contribute to HSC maintenance and expansion via angiocrine CXCL12 and BMP signaling. Middle, human pluripotent stem cell (hPSC)-derived ECs expressing *RUNX1*, *TAL1*, and *GATA2* can differentiate to HSCs. The full differentiation requires cell cycle re-entry. Right, Overexpression of a constitutively active form of the activin receptor-like kinase-2 (*ALK2*) converts HUVECs to adipocytes and chondrocytes. CV: coronary venous, HSC: hematopoietic stem cell, OE: overexpression, HUVECs: human umbilical vein endothelial cells

The opportunity to combine lineage tracing strategies with single-cell transcriptomic approaches provides an opportunity to (re)-investigate developmental pathways and to generate novel mechanistic insights by inferring the developmental history of single cells based on their transcriptional profile [12, 33, 34]. One such example is the re-investigation at single-cell level of coronary artery specification during embryonic development [12]. An accepted view was that blood flow dynamics shape the development of the coronary vasculature [27, 35, 36]. Reconstruction of developmental trajectories of single ECs in the sinus venous using single-cell RNA sequencing showed the existence of a rare type of ECs in veins in the developing murine heart (at E12.5) that can adopt a pre-arterial fate (independently of blood flow dynamics) and eventually differentiates into coronary arterial ECs [12] (Fig. 2A). Of note, in this subset of cells, the expression of *CoupTFII* was decreased, allowing the differentiation of pre-artery cells into arteries. Overexpression of *CoupTFII* blocked the specification of pre-artery cells and their differentiation into arteries (via upregulation of cell cycle genes). This new finding not only adds to our understanding of how artery and vein ECs develop and are inherently plastic, but also may have implications for the treatment of heart disease and the promotion of arterialization [21]. Interestingly, gene fate mapping combined with clonal analysis showed that a subset of embryonic endocardial ECs transition into *Pdgfrα/β*-expressing progenitors, which eventually convert into pericytes and vascular smooth muscle cells (vSMCs) in the heart [37] (Fig. 2B).

### Differentiation of endothelial cells to other cell types

Adult ECs can be reprogrammed to differentiate to competent hematopoietic stem cells (HSCs), a process driven by overexpression of the transcription factors *Fosb*, *Gfi1*, *Runx1*, and *Spi1* [38]. Inductive signals from the vascular niche also contribute to the reprogramming [38]. Indeed, genetically reprogrammed ECs co-cultured in the presence of “vascular niche” cells showed increased self-renewing capacity [38] (Fig. 2B). “Vascular niche” cells were ECs overexpressing *E4ORF1*, which have been shown to maintain an angiogenic phenotype and are capable of HSC maintenance and expansion (Fig. 2B). Inductive angiocrine signals, including CXCL12 and BMP, sustained the expansion and self-renewal of the reprogrammed EC clones. Analysis of single-cell trajectories of the endothelial-to-hematopoietic transition from human pluripotent stem cells (hPSCs) similarly showed that ECs are capable of generating hematopoietic cells in a process governed by *RUNX1*, *TAL1*, and *GATA2* [39]. Here, regulation of cell cycle dynamics appeared critical for the reprogramming into the hematopoietic lineage [39] (Fig. 2B).

In addition to the endothelial/hematopoietic axis, ECs bear the potential to differentiate to cardiac and skeletal muscle cells. Etv2 is a transcription factor expressed by vascular progenitors (including “embryonic ECs”), important for the regulation of the endothelial/ hematopoietic axis [40–42] (Fig. 2B). A single-cell RNA sequencing study of zebrafish embryos showed that loss of *Etv2* precluded the differentiation of endothelial and myeloid cells, while instead ECs acquired alternative fates including cardiomyocytes and a somitic fate via upregulation of myogenic markers [40] (Fig. 2B). In developing chicken limbs, ECs and somites share a common developmental origin [43], and in the mouse, somite Notch signaling promotes a vascular cell-fate choice at the expense of a myogenic fate [44]. In addition, overexpression of a constitutively active form of ALK2 is sufficient to promote the differentiation of ECs into adipocytes and chondrocytes [45] (Fig. 2B). Thus, ECs can diffferentiate to other cell types. Whether such plasticity can be exploited for therapeutic strategies (cell therapy, generation of blood vessel organoids, treatment of malignancies and immunological disorders, etc.) remains to be determined (see Box Exploitation of EC plasticity for regenerative medicine) [46, 47].

## Angiogenic EC phenotypes

### New vessel formation

During embryonic development, numerous vessels are formed from pre-existing ones, in a process called angiogenesis, which can be further divided into sprouting angiogenesis (SA) and intussusceptive angiogenesis (IA) [1, 25, 48]. SA regulates the growth of vessels via sprouting of ECs. In contrast, during IA, pre-existing vessels split and ECs extend inside the vessel and build a new lumen within the pre-existing vessel (via formation of tissue pillars) [49]. This angiogenic mechanism not only allows to grow new vessels via duplication (intussusceptive microvascular growth) but also to remodel the vascular tree via arborization (intussusceptive arborization) and pruning (intussusceptive pruning), contributing to the control of the vascular tree geometry [49]. Angiogenesis in healthy adult organs is rare, with a few exceptions (skeletal muscle angiogenesis during physical exercise; endometrial angiogenesis during epithelial regeneration [50, 51]). However, upon injury, adult ECs can rapidly grow new vessels via re-activation of developmental angiogenic programs (SA or IA).

**Fig. 3:**
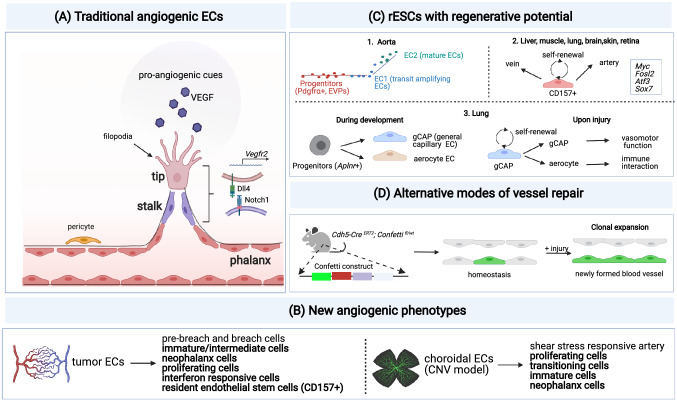
Mechanisms of vessel repair: **A** Illustration of SA, showing the leading tip EC (with filopodia) and stalk ECs and some key molecular regulators of the tip/stalk specification via the VEGF/Notch axis upon pro-angiogenic cues. Tip cells are characterized by high expression of *Dll4* and *VEGFR2* (receptor for VEGF). Dll4 signaling from the tip cells induces the expression of Notch signaling in stalk cells. Quiescent phalanx ECs are indicated. **B** List of EC phenotypes identified by scRNA-seq in models of pathological angiogenesis (tumors; choroidal neovascularization (CNV)). Conserved EC phenotypes across models of CNV and lung cancer [6, 31] are shown in bold. EC phenotypes identified in only one of the two models (tumor or CNV) include breach and pre-breach ECs (tumor-restricted) and shear stress responsive arterial ECs (only in CNV). **C** Scheme of resident endothelial stem cells (rESCs) endowed with regenerative potential: 1. Pseudotime analysis of aortic ECs showing the differentiation trajectories of *Pdgfrα* + endovascular progenitor cells (EVPs) (red line) into two distinct EC subtypes, i.e., EC1 (green top line; transit amplifying ECs) and EC2 (blue bottom line; mature ECs) adapted from 66. 2. CD157 + rESCs (resident endothelial stem cells, capable of self-renewal and differentiation to artery and vein ECs) are found in large vessels of several organs (liver, muscle, lung, brain, skin, and retina). Key transcription factors expressed by CD157 + ECs are listed in the box on the right. 3. Aplnr + progenitor cells in the lungs differentiate into general capillary (gCAP) ECs and aerocyte ECs during embryonic development. Upon lung injury in adult tissues, gCAP ECs are capable of self-renewal and differentiation into aerocytes. gCAP ECs are important in vasomotor control, whereas aerocytes are involved in immune functions (leukocyte trafficking). **D** Clonal expansion represents an alternative mode of vessel repair. Genetic tracing can be used to identify clonal expansion of ECs during vessel repair. Here, mice harboring the Confetti marker were crossed to Cadherin5-Cre ERT2 animals to trace clonal expansion of Cadherin5+ ECs upon injury [109]

**Fig. 4:**
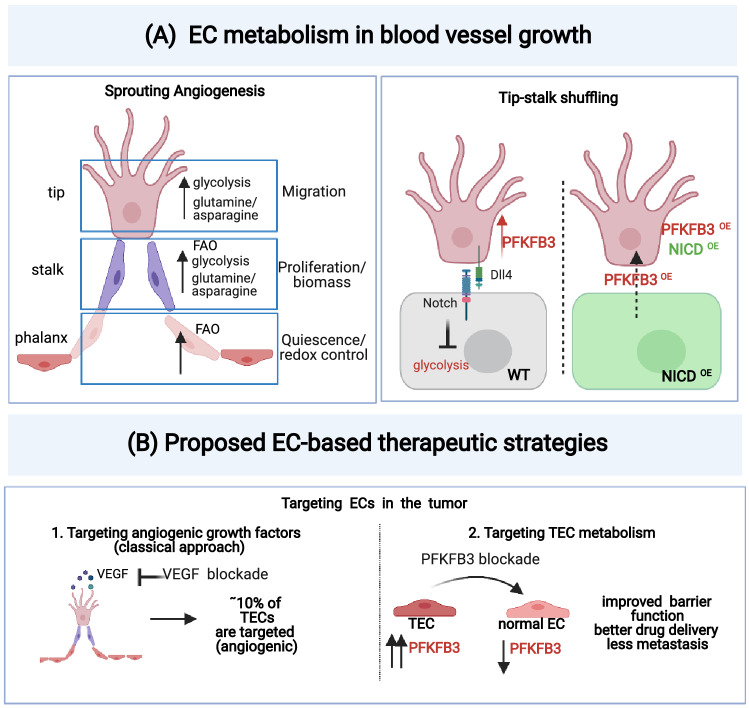
EC metabolism and proposed new EC-targeted therapies: **A** EC metabolism during sprouting angiogenesis: *Left*: Key metabolic pathways associated with each EC phenotype (tip, stalk, and phalanx ECs) involved in sprouting angiogenesis. Glycolysis and amino acid metabolism (glutamine/asparagine) are increased in tip cells to support migration. Fatty acid oxidation (FAO), glycolysis, and amino acid metabolism (glutamine/asparagine) support biomass production in stalk cells, while in phalanx ECs high levels of FAO support the maintenance of the quiescence phenotype (partially via regulation of the redox balance). *Right*: Regulation of tip-stalk cell shuffling: the glycolytic activator PFKFB3 is upregulated in tip cells while Notch signaling inhibits glycolysis in stalk cells. Overexpression of the Notch intracellular domain (NICD) maintains high Notch signaling and the stalk cell phenotype. However, PFKFB3 overexpression is sufficient to turn a stalk cell into a tip cell, even in the case of parallel overexpression of NICD. Therefore, PFKFB3 signaling overrules Notch signaling and drives the tip cell phenotype, and metabolism can fuel vessel sprouting independently of angiogenic signals. **B** Proposed EC-based therapeutic targeting strategies in cancer: 1. Fewer than 10% of tumor ECs are targeted by classic anti-angiogenic therapy (anti-VEGF), since such therapies primarily target angiogenic ECs and not the majority of other tumor ECs, which may partly explain therapy resistance. 2. Targeting of EC metabolism has emerged as a promising approach to target (tumor) angiogenesis. Inhibition of glycolysis via PFKFB3 blockade in tumors resulted in enhanced barrier function, decreased metastasis, and improved drug delivery in animal models

During retinal vascularization, a model of SA has been put forward [52–56]. Angiogenic signals (such as VEGF) induce the formation of a “tip” cell with long filopodia that sense these signals. The tip cell then guides the nascent vessel sprout to the site of VEGF production and at the same time also upregulates the expression of its receptor (*Vegfr2*) (Fig. 3A) [53, 54]. Following the tip cell, proliferating “stalk” cells elongate the sprout (Fig. 3A). When the new vessel becomes perfused, ECs differentiate to quiescent “phalanx” cells. Notably, stalk cells can dynamically differentiate to tip cells and overtake the latter, so that the fittest EC leads the vessel sprout at the forefront [53, 55–57]. Hence, tip and stalk cell phenotypes are not genetically pre-determined, fixed states, but instead represent plastic EC phenotypes (Figs. 3A and 4A).

Single-cell studies of choroidal neovascularization (CNV) and cancer expanded our view on the phenotypic heterogeneity of SA and identified several EC clusters [6, 31]. These include typical angiogenic EC phenotypes (tip and proliferating ECs) and more mature phalanx ECs upon formation of more mature vessels; curiously, typical *bona fide* stalk ECs were not detected, though EC phenotypes expressing some stalk cell features were identified [6, 31]. Newly identified EC subtypes included immature and neo-phalanx ECs [31], and breach cells, i.e., an EC subpopulation sharing gene markers with tip cells, but also expressing genes involved in podosome rosette formation to prepare for/assist tip cell migration [6] (Fig. 3B). By contrast, to date, nearly nothing is known about the molecular and cellular heterogeneity of ECs during IA.

Recent single EC transcriptomic studies using pseudotime analysis enabled in silico lineage tracing of ECs during SA. These analyses revealed that, in neovascular diseases, post-capillary venule ECs initiate vessel sprouting and then progress over intermediate states to differentiate to tip cells and subsequently to more quiescent phalanx-like ECs, once vessel formation has been established [31]. Of note, cells expressing genes associated with resident endothelial stem cells (rESCs) were enriched in post-capillary venules in tumors [6] (Fig. 3B), raising the question whether they might contribute to vessel formation and regeneration, as shown previously [58, 59]. Notably, since the venous EC population (containing rESCs) expanded upon anti-VEGF treatment of tumor-bearing mice, these findings raise the question whether an enlarged pool of rESCs may contribute to the resistance against anti-VEGF therapy.

During development, Notch and VEGF regulate arterial fate and vessel sprouting [60, 61]. In SA, VEGF induces the migration of tip cells and the expression of Dll4. Dll4 activates Notch in stalk cells, impairing acquisition of the tip phenotype [53, 54] (Fig. 3A). Depending on the level of Notch expression, ECs may sprout or differentiate to arteries while exiting the cell cycle. On the contrary, COUP-TF2 inhibits the arterial fate and at the same time upregulates cell cycle genes [23, 26, 62]. Of note, the interplay between cell-fate decisions, angiogenesis, and cell cycle has been re-investigated. Using a combination of bulk transcriptomic and fate mapping approaches in mosaic mice with heterogeneous Notch expression [28], Notch, rather than directly regulating the expression of arterial fate genes, inhibits the transcriptional activity of Myc and downstream cell cycle and metabolic genes, suggesting a role for cell cycle and metabolic genes in the regulation of SA and EC identity [28].

### Vessel repair

ECs are continuously exposed to circulating pathogens, toxic substances, and hazardous agents and therefore can undergo damage, requiring continous repair/regeneration. Also, diseases such as diabetes, atherosclerosis, and others are characterized by EC dysfunction [63]. Notably, single-cell RNA sequencing of ECs revealed a small number of angiogenic ECs in healthy quiescent tissues [4], raising the question whether they might be involved in new vessel formation or regeneration of dysfunctional ECs. In agreement, analysis of the EC renewal rate in the heart of individuals between 20 and 70 years of age revealed several cycles of EC turnover throughout adulthood [59, 64].

With regard to the mechanisms of vascular repair, a matter of debate is whether different EC subtypes with stem-like features exist in all or only in large vessels and if additional (non-EC) cells may participate in this process [58, 65–70]. Two single-cell studies identified possible novel mechanisms of EC regeneration [58, 65]. Both studies showed that vascular regeneration is largely driven by vessel wall-resident ECs, therefore excluding (at least in the used model systems) a major contribution by circulating stem or hematopoietic progenitors [69–72]. In one study, regeneration of the endothelium relied on a relatively rare population of CD31 + /CD45- rESCs, enriched for the expression of the surface markers CD157 and CD200 and for several transcription factors including *Myc*, *Fosl2*, *Atf3*, and *Sox7* and detected in large vessels of several organs (liver, heart, lung, intestine, skin, muscle, and brain) [65] (Fig. 3C). In two different models of tissue repair (liver regeneration, hindlimb ischemia), CD157+ ECs were capable of repairing the vessel via activation of an angiogenic program [65]. In a second report, repair of the aorta was driven by ECs and not by circulating progenitors [58]. ECs close to the site of injury were responsible for regeneration of the damaged vessel and the gene expression profile of these regenerating ECs did not resemble that of sprouting ECs [58]. In fact, these regenerating ECs show a gene signature indicative of a coordinated and rapid switch from quiescence to highly proliferative cells with genes associated with maintenance of cell junctional complexes. Moreover, genes associated with migratory behavior were absent in these cells. Also, single-cell analysis identified quiescent endovascular progenitors (EVPs) in the aorta, characterized by a high mitochondrial content (as observed in other stem cells/progenitors) [66, 73, 74], enriched in mesenchymal markers (such as PDGFR-*α*), which transitioned to two differentiated EC populations (transit amplifying ECs and mature ECs) upon pseudotime trajectory analysis [66] (Fig. 3C). Thus, vessel wall-resident EC progenitors may contribute to repair of damaged ECs.

In addition to large vessels, the microvasculature (in lungs) may also harbor stem/progenitors for ECs [67]. During embryonic development, *Aplnr* + progenitor cells in the lungs differentiate into general capillary (gCAP) ECs, which are responsible for vasomotor control and aerocytes (lung-specific ECs important for leukocyte trafficking). In adult tissues, gCAP ECs themselves were capable of self-renewal and differentiation into aerocyte ECs during lung injury, demonstrating their stem/progenitor-like features [67, 75] (Fig. 3C). Similarly, a subpopulation of capillary ECs with stem-like features exists in the microvasculature of lymphoid tissues [68].

## EC heterogeneity in cancer

Angiogenesis is a hallmark of cancer. Earlier studies using bulk or targeted approaches revealed that tumor ECs (TECs) differ from their normal counterparts and exhibit considerable heterogeneity [22, 76–79]. For instance, single-cell RNA sequencing and comparative microarray analysis revealed that TECs from human lymphoma, breast, and colorectal cancer display a distinct gene expression signature compared to their healthy counterparts, enriched in genes involved in extracellular matrix (ECM) metabolism and collagen formation, vascular smooth muscle contraction, and signaling pathways [80].

Recent single-cell studies expanded these insights considerably. Single-cell analysis of human non-small cell lung cancer (NSCLC) discovered distinct transcriptome signatures in TECs compared to ECs from non-malignant lung tissue [81], with Myc targets presenting the top upregulated pathway in TECs, in line with Myc’s role in tumor angiogenesis [82]. Surprisingly, genes involved in antigen presentation (MHC class I and II), chemotaxis (*CCL2*, *CCL18*, *IL6*), and immune cell homing (*ICAM1*) were downregulated in TECs compared to normal lung ECs, raising the question whether TECs potentially contribute to immune tolerance [81]. In agreement, compared to early-stage ground glass nodules adenocarcinoma (GGN-ADC), TECs from late-stage solid adenocarcinoma (SADC) exhibited lower levels of genes involved in antigen presentation and chemotaxis [83].

Follow-up single-cell RNA sequencing of isolated ECs from paired human NSCLC and peri-tumoral tissue and from murine lung tumors and healthy lungs provided additional understanding [6]. These studies together identified 33 EC phenotypes in tumor and healthy lung tissue and yielded the following insights [6]. First, fewer than 10% of TECs in human NSCLC were angiogenic tip or proliferating ECs, raising the question whether this contributes to the resistance of VEGF-blockade anti-angiogenic therapy and can explain its relative insufficient efficacy [6] (Fig. 4B). Traditional stalk cells were not detected [6]. Second, capillary ECs in healthy lung expressed a gene signature of semi-professional antigen-presenting cells, but these immunomodulatory ECs are underrepresented in lung tumors [6]. In agreement, genes related to antigen presentation and chemotaxis were downregulated in TECs from SADC compared to early-stage GGN-ADC [83]. On the other hand, ECs with transcriptomic features of high endothelial venules (HEVs) were more abundant in tumors [84], suggesting a complex regulation of tumor immunity by TECs. Third, breach TECs were identified, likely assisting tip cell migration (Fig. 3B, see above) [6]. Fourth, upon treatment of tumor-bearing mice with VEGF-targeted compounds, tip and breach TECs were most sensitive, proliferating TECs were not more sensitive (likely indicating that other angiogenic signals were driving TEC proliferation), while venous TECs were less sensitive [6]. In agreement, venous TECs expanded upon VEGF-blockade therapy. Given that venous ECs contained rESCs (see above), it remains to be determined whether any possible expansion of this population might contribute to the resistance against VEGF-blockade therapy [6].

Generating long lists of gene markers for distinct cell populations by single-cell RNA sequencing (scRNA-seq) is becoming a standard procedure; however, utilizing this data tsunami to identify and prioritize new angiogenic targets is a formidable challenge. One study hypothesized that EC phenotypes or gene markers, congruently upregulated across species, diseases, tissues, and experimental conditions, would represent biologically more important candidates and therapeutically more attractive targets [6]. An integrated multi-disciplinary approach, involving scRNA-seq, complemented with validation bulk transcriptomics and proteomics studies, illustrated the potential of this approach for identifying new angiogenic targets, which were functionally validated in vitro and in vivo [6] (Box Novel integrated analysis to identify & validate scRNA-seq targets).

A limitation of current research on EC heterogeneity in cancer is that still very little is known about alternative modes of tumor vessel formation, including vessel splitting (IA) [85, 86] and vessel co-option [87]. Nonetheless, tumors may switch from SA to vessel splitting or co-option when treated with VEGF blockade [85, 88, 89]. These alternative tumor vascularization modes may thus help escape the tumor from and cause resistance to traditional anti-angiogenic therapy.

## EC heterogeneity in tissue repair

In 2019, a new strain of Coronavirus, severe acute respiratory syndrome (SARS)-CoV-2, caused a global pandemic. COVID-19, the disease caused by SARS-CoV-2 infection, is characterized in severe cases by life-threatening acute respiratory distress syndrome (ARDS) in the lungs and by widespread multi-organ failure [90]. A perspective hypothesized that EC dysfunction contributed to COVID-19 vascular complications (vascular leakage, thrombosis, inflammation) [90–95]. Also, vessel splitting (IA) appeared to be more prevalent in COVID-19 lungs [92, 93]. Even though neovascularization is a common denominator of other lung diseases (acute lung injury [96], idiopathic pulmonary fibrosis [97]), it remains elusive which particular EC subtypes are involved. We will highlight a few examples of the role of ECs in tissue repair and alternative EC functions, which have recently become more evident.

First, ECs may modulate immune responses as “immunomodulatory ECs (IMECs)” [4, 98, 99]. Analysis of ribosome-bound mRNAs (reflecting functionally relevant transcriptome changes) of ECs in three different tissues (lung, heart, brain) revealed that inflammation (induced by LPS) resets ECs with a broad representation of genes involved in leukocyte/immune cell trafficking [99]. Notably, LPS-treated ECs from different tissues transcriptomically resembled each other more closely than untreated ECs [99]. In single-cell studies, EC subsets have been identified expressing genes involved in scavenging, immunoregulation, immune surveillance, and interferon signaling across different tissues [4, 8] (Fig. 3B). Identification of markers to discriminate these subtypes coupled with functional studies will be important to address which exact immunomodulatory function (pro- *versus* anti-inflammatory) these EC subsets have.

Of note, scRNA-seq and ATAC-sequencing (to characterize the epigenomic landscape) of ECs from mouse carotid arteries after partial ligation illustrated that disturbed flow induced reprogramming of ECs toward an immune cell-like phenotype (coined “EndICLT”), expressing macrophage markers [100]. Tissue-specific endothelial genes (enriched in the brain, heart, lungs, and kidney [4]) were expressed at low or undetectable level in carotid ECs [100], further illustrating the tissue- and vascular bed-specific EC heterogeneity. Notably, disturbed flow also induced this EndICLT phenotype in cultured human aortic ECs, in the absence of immune cells [100]. Thus, ECs are highly plastic cells that transition to immunomodulatory cell phenotypes, identifiable by single-cell analysis as a separate subset [100]. ECs have been previously involved in immune responses [101, 102]. Such phenotypic plasticity of ECs to IMECs is not unique to ECs, since epithelial cell-to-immunomodulatory cell transition has been reported [103].

Multi-omic approaches (epi-genomic atlases; spatially resolved gene expression) allow to generate in-depth mechanistic insights [104, 105]. In one such study, simultaneous bulk analysis of the transcriptome and epigenome of ECs across 12 organs showed that ECs are epigenetically pre-programmed for a response to immunological challenges, including viral infection [106]. Future studies will inform on the contributions of distinct IMEC subsets to immunity in normal tissue homeostasis, tumor immunity, and graft rejection reactions [107, 108].

Second, vascular repair in a model of myocardial infarction does not need to rely on SA alone, but can also occur via alternative vascularization modes [109, 110]. For instance, use of the Confetti reporter mouse line crossed with *Cadherin 5*-Cre mice (to enable clonal analysis of Cadherin5+ ECs) revealed that vessel repair can proceed via clonal expansion of proliferative clusters of ECs as determined by the analysis of the fluorescence color distribution of the labeled cells after myocardial injury [109] (Fig. 3D). Such proliferative EC subtypes (characterized by enriched expression of cell cycle and metabolic genes) have also been described in a model of choroidal SA [31]. In addition to SA (which requires EC proliferation), IA represents an alternative mode of vascularization for tissue repair. Indeed, for instance in the lung, SA is detected in usual interstitial pneumonia (UIP) [111] and H1N1-infected lung explants [92], while IA is documented in rat models of glomerulonephritis [112] and colitis [113], in lungs of patients affected by nonspecific interstitial pneumonia (NSIP), alveolar fibroelastosis (AFE) [111], and COVID-19 disease [92].

Third, vascular repair may involve the re-appearance of a known EC subtype in a different location. For instance, in healthy lungs, peri-bronchial ECs are restricted to the bronchial vasculature in large airways, while in idiopathic pulmonary fibrosis (IPF), peri-bronchial ECs (characterized by high *COL15A1* expression) were enriched in IPF lungs, in particular in areas of bronchiolization and fibrosis [114]. This is likely a compensatory mechanism, whereby destruction of peripheral pulmonary capillaries leads to an increase in pulmonary pressure with hypertrophy and neo-angiogenesis of peribronchial vessels.

## The role of metabolism in EC plasticity and heterogeneity

When angiogenic signals stimulate vessel formation, ECs must adapt their metabolism in order to produce the energy and biomass and to secure redox homeostasis, necessary for ECs to divide, grow, and migrate during vessel formation [63, 115]. A key transgenic experiment provided the first proof-of-evidence for the essentiality of EC metabolism, illustrating that EC metabolism could even overrule the activity of angiogenic signals [116] (Fig. 4A). As explained above, tip and stalk cells are interchangeable EC phenotypes, with stalk cells overtaking the leading tip position in a highly dynamic process regulated by Notch (high Notch signaling promotes the stalk cell phenotype) [54, 55]. Remarkably, in vivo evidence from a zebrafish SA model showed that over-expression of the glycolytic activator PFKFB3 is sufficient to convert a stalk cell into a tip cell, despite high Notch signaling [induced by the overexpression of the Notch intracellular domain (NICD)], thus overruling Notch-induced stalk cell conversion [116] (Fig. 4A). Conversely, in an in vitro SA model using mosaic EC spheroids, *PFKFB3* gene silencing impaired the tip cell competitiveness [115–117]. Of note, low levels of glycolysis in stalk cells contribute to proliferation in these cells (Fig. 4A).

Notably, different metabolic enzymes are capable of driving EC plasticity. Just to give a few examples, carnitine palmitoyl-transferase 1A (CTP1A), a rate-controlling enzyme of fatty acid oxidation (FAO), results in the production of acetyl-CoA to sustain (in conjunction with anaplerotic substrates) the tricarboxylic acid (TCA) cycle to stimulate nucleotide synthesis in proliferating ECs [118], but this enzyme also maintains redox homeostasis in quiescent ECs [119]. Thus, FAO is critical for biomass production in stalk ECs and for quiescence in phalanx ECs during angiogenesis (Fig. 4A). At the same time, FAO promotes venous-to-lymphatic EC differentiation by increasing histone acetylation at lymphangiogenic genes, thereby increasing the expression of the lymphatic master transcription factor *Prox-1* [120, 121]. Other metabolic pathways fuel the differentiation of tip cells and stalk cells. For instance, glutamine and asparagine metabolism is essential for stalk-to-tip conversion during SA [122] (Fig. 4A), while fatty acids and ketone bodies promote EC proliferation in blood vessels and lymphatics [123, 124]. When differentiating to ECs, stem/progenitor cells in the mouse aorta increase glycolysis and oxidative metabolism, a mechanism possibly regulated by AKT/ mTOR signaling [125]. In addition, FOXO1 regulates EC quiescence by suppressing Myc and cellular metabolism [126], while the pro-quiescence transcription factor KLF2 suppresses glycolysis in ECs [127]. Fatty acid synthase (FASN), a critical enzyme in lipid synthesis, proved important for EC proliferation and vessel sprouting through malonylation of mTOR [128]. Another enzyme affecting EC function is the M2 isoform of pyruvate kinase (PKM2), which is important for proliferating ECs to ensure cell cycle progression (via inhibition of NF-κB/p53 signaling) [129, 130] and in quiescent ECs to maintain vascular barrier function. Intriguingly, PKM2 has also been involved in modulating EC immune functions via epigenetic control (indirectly by impacting S-adenosylmethionine synthesis) of anti-viral innate immune signaling of ECs [129]. For a more detailed description of metabolic regulation of EC biology, we refer to comprehensive reviews [19, 121, 131].

Single-cell studies revealed a greater metabolic transcriptome heterogeneity than expected. Indeed, when focusing only on the expression of metabolic genes by ECs from different healthy mouse tissues, each EC subtype in each different tissue could be distinguished by a particular metabolic gene signature [4] (Fig. 1B). This was also the case for ECs in diseases, characterized by new vessel formation (tumors in the lung, CNV in the eye) [6, 31]. Notably, ECs from some tissues expressed largely similar metabolic transcriptomes, presumably because they share biological features (see above), suggesting an important contribution of the metabolic transcriptome to the EC identity [4, 31]. In addition, ECs from different tissues exhibited metabolic transcriptome heterogeneity. For example, brain ECs have a higher expression of glucose and amino acid transporters (*Glul*, *Slc2a1*, *Slc6a6*), while splenic ECs show gene expression related to cholesterol metabolism (*Cyp51*, *Sqle*, *Idl1*) [4, 19]. In turn, cAMP metabolic gene expression is elevated in lung ECs (*Pde9a*, *Pde3a*, *Pde3b*), while cardiac and muscle ECs exhibit enrichment of genes involved in lipid uptake and metabolism (*CD36*, *Pparg*, *Lpl*) [4, 132] (Fig. 1B). Even more, ECs display metabolic gene signature heterogeneity within the vascular tree, in a tissue type-specific pattern. Indeed, in the liver, ECs from each vascular bed (arteries, capillaries, veins) express distinct metabolic gene markers, while, in the brain, ECs from these vascular beds share common markers or express vascular bed-specific markers [4]. In agreement, ECs from certain vascular beds in the brain showed heterogeneity in the expression of transporter genes [10, 99, 133]. Renal ECs also exhibit zone-dependent metabolic transcriptome heterogeneity. Indeed, compared to glomerular and cortical ECs, medullary ECs were enriched in genes involved in oxidative phosphorylation (OXPHOS), a finding validated by metabolic flux analysis [9]. OXPHOS was essential for medullary ECs to cope with dehydration stress [9].

Compared to normal ECs, proliferating ECs in murine lung tumors and in CNV showed increased expression of genes involved in glycolysis, one-carbon metabolism, nucleotide synthesis, TCA cycle, and OXPHOS [31]. Even despite the importance of glycolysis for ECs [63, 116, 117], OXPHOS is required for nucleotide production during in vivo angiogenesis [134, 135]. Several metabolic adaptations were detected in subsets of TECs in NSCLC. To name a few, genes involved in lipid metabolism are upregulated in capillary TECs, while venous TECs exhibit an increased prostaglandin metabolic transcriptome signature [31]. Further, compared to early-stage GGN-ADC, TECs from late-stage SADC are enriched in metabolic processes [83]. Comparative single-cell analysis of three distinct cancer types (colorectal, lung, ovarian) revealed upregulation of genes involved in glycolysis and OXPHOS in tip cells [84]. Compared to capillary ECs in normal lungs, TECs in lung tumors downregulated the expression of carbonic acid metabolism (characteristic of alveolar ECs), but instead enhanced the expression of genes involved in glycolysis and OXPHOS [84]. Using an EC-tailored genome-scale metabolic model as part of an integrated multi-omics analysis, *Aldh18a1* and *Sqle* were identified as consistently upregulated genes in angiogenic ECs in tumors and CNV and demonstrated to be critical for EC proliferation, migration, and vessel sprouting [31].

## Perspectives and therapeutic implications

Historically, ECs were categorized according to their anatomical position in the vascular tree (e.g., arteries, capillaries, veins) and, more recently, in the vessel sprout (e.g., tip, stalk, phalanx cells). Bulk-omics analyses revealed that ECs exhibited tissue-specific gene signatures. Single-cell analyses, capturing transitory functional states no longer based only on their position in the vascular tree, now reveal that ECs are more plastic and heterogeneous than anticipated. For instance, ECs in a healthy vessel can flexibly adapt to change their transcriptome so that they temporarily differentiate to a new EC phenotype (such as immuno-modulatory ECs), which nonetheless can be identified as a statistically separable phenotype/EC subtype. The evolution to consider these functional EC phenotypes as distinct EC phenotypes is unstoppable, and within the near foreseeable future, these functional EC phenotypes will become targets for new vascular oriented therapies, though it will be challenging to decipher how to target particular EC phenotypes. Given that these EC phenotypes can exist transiently, an option might be to consider strategies to “retune” such functional phenotypes, rather than the “pruning” approach of past/current anti-angiogenic therapy. On the other hand, progress has been made regarding the targeting of different metabolic pathways in ECs to inhibit angiogenesis. Inhibition of PFKFB3 in a pre-clinical model of lung cancer reduced angiogenesis, decreased metastasis, enhanced barrier function, and improved drug delivery [116, 117, 136] (Fig. 4B).

Another major challenge is to identify and to prioritize, among the multiple possible candidates identified by single-cell analyses, the true targets for further clinical translation. Combining single-cell approaches with congruent gene expression analysis across multiple species, tissues, conditions etc. is a powerful strategy to identify biologically relevant targets (Box Novel integrated analysis to identify & validate scRNA-seq targets). Multi-omics approaches combining spatial and epigenetic information with transcriptomic signatures promise to further provide an additional strategy to better address the mechanisms that govern this heterogeneity [104].

Information boxes

Box 1: Exploitation of EC plasticity for regenerative medicine ECs display great plasticity exemplified by their ability to differentiate into numerous EC subtypes and their trans-differentiation into distinct cell lineages, such as hematopoietic stem cells (HSCs) and cardiomyocytes. This plasticity might offer an opportunity to use ECs in clinical applications, in particular in the field of regenerative medicine.

Engineering blood vessels ex vivo Ex vivo EC organoid systems are used to study vascular development and neovascularization, to mimic in vivo drug treatments, as well as for cell therapy and transplantation. An established method to create blood vessel organoids is the derivation of ECs from human pluripotent stem cells (hPSCs) in 3D models [137, 138]. ECs can be derived from hPSCs by induction of mesodermal fate and subsequent specification of the vascular fate [139]. One exciting application of hPSC-induced blood vessel organoids is the construction of vascularized tissue grafts [140]. A major challenge in tissue transplantation is the integration of the graft into the recipient’s vasculature. Astonishingly, hPSC-derived blood vessel organoids cannot only form fully functional networks in vitro, but also integrate with the host vasculature in vivo, as demonstrated in a model of diabetic vasculopathy, where long-term engraftment was achieved in mice [141].

Cell transfer therapies Several pathologies are caused or accompanied by vascular dysfunction and chronic ischemia. Autologous use of ECs in such contexts might offer therapeutic opportunities. For instance, transplantation of CD157+ progenitor ECs can promote endothelial regeneration and new vessel formation [46]. Moreover, as explained above, ECs can be generated from hPSCs for potential cell therapy purposes. The discovery of specialized EC subtypes by single-cell studies also raises the question whether hPSCs-derived ECs can be tuned to phenotypes, which favor angiogenesis. On the other hand, ECs are not only capable of transitioning between different states, but can also transdifferentiate into distinct cell lineages. Several protocols have been developed to derive different cells from ECs, including HSCs. Here, the mechanistic insights gained from studies investigating blood cell formation from ECs during embryogenesis (a process known as endothelial-to-hematopoietic transition (EHT)) allowed successful transformation of adult ECs from different organs into fully competent HSCs. While the utilization of such EC-derived HSCs in the clinical setting has yet to be tested, these findings hold possible clinical potential for transplantation and cell therapies.

Box 2: Novel integrated analysis to identify & validate scRNA-seq targets Most transcriptomic analyses of ECs differ in study design with respect to species (human vs. mouse) and controls for the pathological setting (healthy ECs from the same organ, healthy ECs from a different organ; and in case of tumors: ECs from peritumoral tissue or ECs from different tumor types). These differences between studies make it challenging to identify common EC subtypes with robust transcriptomic signatures. In addition, prioritization of targets is a formidable challenge when interpreting large datasets. To overcome these challenges, integrated multi-disciplinary approaches (involving scRNA-seq, complemented with proteomics, bulk transcriptomics, and functional studies) or multi-omics approaches (e.g., transcriptomic studies with epi-genomic atlases and/or spatially resolved gene expression studies) are emerging new tools that can facilitate the identification of robust transcriptomic EC phenotypes and/or provide mechanistic insights that govern EC heterogeneity. Here, we provide a brief overview of novel multi-omics and integrated analyses that recently emerged to analyze scRNA transcriptomic data with the goal to identify meaningful targets.

Orthogonal multi-omics and meta-analysis of scRNA-seq data This approach was recently used to reconstruct TEC taxonomies in lung cancer, using human (4 different lung tumor subtypes) and mouse (orthotopic model of lung cancer) tissues to identify new tumor-specific EC subtypes not present in healthy tissues. A congruent meta-analysis was performed to identify conserved TEC phenotypes and angiogenic pathways (putative new targets) across these different species (human and mouse) and models, including on top of the above-mentioned datasets, cultured TECs as well as interrogation of publicly available bulk transcriptomics datasets. To further increase the power of target predictions, a meta-analysis of 144 bulk proteomic datasets was performed which, in combination with the transcriptomic data, led to the identification of highly conserved (and therefore likely to be functionally relevant) angiogenic targets, among which were genes involved in collagen hydroxylation [6]. Such an integrated approach is becoming increasingly popular and several computational strategies are available to perform such integrated analysis across large datasets [142].

Integration of genome-scale metabolic models (GEM) with scRNA-seq data ScRNA-seq studies have been used to profile metabolic gene signatures at the single-cell level [4, 6, 31]. However, transcriptomic studies do not provide insight in metabolic flux activities. GEnome-scale Metabolic models (GEMs) are mathematical computational models of active metabolic reactions, which can be tailored to a particular cell type by integrating multi-omics and other data and can be used to predict the essentiality of particular metabolic reactions. Combining scRNA-seq analyses with the use of an EC-tailored GEM (Endo-GEM) offers the opportunity to prioritize metabolic targets. Indeed, in one study [31], scRNA-seq data were first generated from two models of pathological angiogenesis (choroidal neovascularization (CNV) and tumor angiogenesis) and then integrated with Endo-GEM to identify EC-specific metabolic signatures, congruently upregulated in both models. Using Endo-GEM, 288 essential genes were identified, including genes previously shown to be important for EC metabolism (glycolysis, OXPHOS, fatty acid oxidation, nucleotide synthesis, and salvage), but also genes involved in cholesterol biosynthesis, sphingolipid metabolism, and amino acid metabolism, previously unknown to be essential for vessel formation [31]. Using an integrated approach, new angiogenic target genes (*i.e., Sqle* and *Aldh18a1*) were identified and functionally validated [31]. Of interest, this integrative approach has been recently applied also to other diseases [143].

Multi-omics platforms Emerging multi-omic platforms offer the opportunity to simultaneously profile RNA and DNA and/or protein at the single-cell level and with spatial resolution. Recent examples include scRNA-seq and chromatin accessibility analysis of ECs in a mouse model of disturbed flow [100] as well as the analysis of the chromatin landscape of cardiac ECs [144] and brain ECs [145]**.** Spatial analyses are also being used to map (endothelial) cell heterogeneity across tissues and conditions including in large cohorts of tissues [146].
